# Co/ZnO/Nitrogen-Doped Carbon Composite Anode Derived from Metal Organic Frameworks for Lithium Ion Batteries

**DOI:** 10.3390/polym14153085

**Published:** 2022-07-29

**Authors:** Ya-Chun Chang, Chia-Hung Huang, Wei-Ren Liu

**Affiliations:** 1 Department of Chemical Engineering, R&D Center for Membrane Technology, Research Center for Semiconductor Materials and Advanced Optics, Chung Yuan Christian University, Taoyuan City 320, Taiwan; ziv910543@gmail.com; 2 Department of Electrical Engineering, National University of Tainan, Tainan City 700, Taiwan; chiahung@mail.mirdc.org.tw; 3 Metal Industries Research and Development Centre, Kaohsiung 701, Taiwan

**Keywords:** zeolitic imidazolate framework-8, zeolitic imidazolate framework-67, lithium ion batteries, anode

## Abstract

Through high-temperature sintering and carbonization, two Co/ZnO nitrogen-doped porous carbon (NC) composites derived from ZIF-8 and ZIF-67 were manufactured for use as anodes for Li ion batteries: composite-type Co/ZnO-NC and core-shell-type Co@ZnO-NC. X-ray diffraction analysis, scanning electron microscopy, and the Brunauer–Emmett–Teller (BET) method were performed to identify the pore distribution and surface morphology of these composites. The findings of the BET method indicated that the specific surface area of Co/ZnO-NC was 350 m^2^/g, which was twice that of Co@ZnO-NC. Electrochemical measurements revealed that Co@ZnO-NC and Co/ZnO-NC had specific capacities of over 400 mAh g^−1^ at a current density 0.2 A g^−1^ after 50 cycles. After 100 cycles, Co/ZnO-NC exhibited a reversible capacity of 411 mAh g^−1^ at a current density of 0.2 A g^−1^ and Co@ZnO-NC had a reversible capacity of 246 mAh g^−1^ at a current density of 0.2 A g^−1^. The results indicated that Co/ZnO-NC exhibited superior electrochemical performance to Co@ZnO-NC as a potential anode for use in Li ion batteries.

## 1. Introduction

With developments in technology, the number of products such as smartphones, digital cameras, and laptops is increasing. Thus, energy storage devices with a small size, a light weight, and a high performance are required [1]. Among rechargeable batteries, lithium ion batteries (LIBs) have attracted considerable attention due to their low raw material cost, high capacity, high battery voltage, long cycle life, and excellent stability at high temperatures [2,3]. LIBs are internationally recognized as the most favourable chemical energy source available. Most LIBs used in commercial applications employ carbon materials, such as graphite, as anode materials. However, the theoretical capacity of graphite anode materials is only 372 mAh/g, which is not adequate for use in LIBs [4,5,6]. Thus, it is urgent to develop higher reversible capacity anode materials for Li ion batteries.

Recently, metal organic frameworks (MOFs) have become increasingly popular because of their advantages of easy preparation, high porosity, high specific surface area, and adjustable pore size [7,8]. Metal–organic frameworks (MOFs) are fabricated by linking inorganic and organic units by weak bonds (reticular synthesis), which form porous materials with a periodic network structure through self-assembly under the actions of coordination bonds [9,10,11,12]. Due to the simplicity and low cost of preparing MOFs, they have considerable potential for future applications, including gas storage [10,11,12,13,14,15,16], drug delivery [17,18,19,20,21], molecular separation [22,23,24,25], chemical catalysis [26,27,28,29,30,31], and energy storage [32,33,34,35,36,37,38,39,40,41,42,43,44]. According to Tai et al.’s work, the as-synthesized ZIF-8 calcined at 800 °C under an N_2_ atmosphere and converted to N-doped carbon can be used as an anode for LIBs. The reversible discharge capacity of 440.5 mA g^−1^ at 1 A g^−1^ is over 100 cycles [35]. Son et al. synthesized ZIF-8 under N_2_ flow and impregnated in thiourea and ethanol solution; the material was then calcined under N_2_ at 900 °C. Finally, they synthesized N, S-doped nanocarbon, which exhibited high electrochemical performance [36]. Tang et al. proposed ZIF-8@ZIF-67 core-shell nanostructures by using a seed-mediated growth technique [37]. Cheng et al. calcined ZIF-8@ZIF-67 in a tube furnace at 600 °C under the N_2_ atmosphere and then subjected it to oxidation in a muffle furnace at 350 °C to obtain ZnO/Co_3_O_4_/N-doped carbon nanocages as anode materials for LIBs. The composite exhibited a reversible capacity of 1305 mAh g^−1^ at 0.1 A g^−1^ after 300 cycles [38]. Huang et al. subjected porous Co-Zn/N-C polyhedral nanocages to carbonization under the Ar atmosphere at 800 °C and used the resulting materials as anodes for LIBs; the materials exhibited a reversible capacity of 702 mAh g^−1^ at 0.1 A g^−1^ after 400 cycles and exhibited satisfactory cycling stability [39]. Zhao et al. demonstrated a ZnO/Co_3_O_4_/CoO/Co composite derived from ZIF-8 and ZIF-67 as precursors and subsequently calcined under the N_2_ flow at 600 °C and then at 200 °C in air atmosphere. The ZnO/Co_3_O_4_/CoO/Co composite anode exhibit a high reversible capacity and excellent cycling performance for Li ion batteries [40].

In this study, we propose two different kinds of Co/ZnO/nitrogen-doped carbon composite anode via two synthesis methods to investigate the structure and electrochemical behaviour of anode materials for Li ion batteries. In the first method, ZIF-8 and ZIF-67 precursors were mixed and stirred to produce ZIF-8/ZIF-67. In the second method, ZIF-8 powder was added to ZIF-67 precursors and stirred to produce ZIF-8@ZIF-67. After high-temperature treatment and carbonization, two samples (Co/ZnO-NC and Co@ZnO-NC) were synthesized as anode materials for LIBs. Tests were then conducted on the samples, including X-ray diffraction (XRD), scanning electron microscopy (SEM), Brunauer–Emmett–Teller (BET) analysis, charge/discharge tests, cyclic voltammetry (CV), and A.C. impedance. The results indicated that the as-synthesized Co/ZnO-NC composite anodes have potential use as anode materials for LIBs.

## 2. Materials and Methods

### 2.1. Synthesis of ZIF-8

In total, 1.89 g of Zn(NO_3_)_2_·6H_2_O (98%, Alfa Aesar, MA, USA) was added to 50 mL of methanol (99%, TEDIA, Fairfield, CT, USA), and 3.92 g of 2-methylimidazole (99%, ACROS, NJ, USA) was then dissolved in 25 mL of methanol. The two solutions were mixed, followed by magnetic stirring for 24 h at room temperature to obtain a mixing solution. The resultant precipitate was collected through centrifugation and washed with methanol three times. Finally, the precipitate was dried at 80 °C for 24 h.

### 2.2. Synthesis of the ZIF-8@ZIF-67 Composite

In total, 0.42 g of ZIF-8 was added to 25 mL of methanol (solution A). Simultaneously, 1.98 g of Co(NO_3_)_2_·6H_2_O (98%, SHOWA, Tokyo City, Japan) was added to 50 mL of methanol (solution B), and 3.98 g of 2-methyl imidazole was dissolved in 25 mL of methanol (solution C). Subsequently, solutions B and C were simultaneously poured into solution A. The mixed solution was stirred for 24 h at room temperature and then collected through centrifugation and washed thrice with methanol. The solution was then dried in an oven at 80 °C for 24 h, and a purple powder was obtained.

### 2.3. Synthesis of the ZIF-8/ZIF-67 Composite

In total, 7.9 g of 2-methyl imidazole was dissolved in 100 mL of methanol. Subsequently, 1.89 g of Zn(NO_3_)_2_·6H_2_O and 1.98 g of Co(NO_3_)_2_·6H_2_O were simultaneously added to the solution. The mixed solution was stirred for 24 h at room temperature to obtain a mixing solution. The resultant precipitate was collected through centrifugation and washed thrice with methanol. Finally, the precipitate was dried at 80 °C for 24 h to obtain the requisite sample.

### 2.4. Synthesis of Co/ZnO-NC and Co@ZnO-NC Composites

ZIF-8/ZIF-67 and ZIF-8@ZIF-67 samples were calcined at 800 °C under the Ar atmosphere at a heating rate of 5 °C min^−1^. Finally, the as-synthesized Co/ZnO-NC and Co@ZnO-NC composites were obtained.

### 2.5. Characterizations

Co/ZnO/nitrogen-doped carbon composite was tested through XRD (Bruker D8 Advance Eco, Karlsruhe City, Germany) by using Cu K_α_ radiation (λ = 0.15418 nm). XRD data were collected in the range of 5° to 80° at a scan rate of 5°/min. X-ray photoelectron spectroscopy (XPS; Thermo Fisher Scientific, Waltham, MA, USA) was conducted using an X-ray photoelectron spectrometer with monochromated Al K radiation. The specific surface area and the pore distribution of samples were determined using the BET method (Micromeritics ASAP-2020, Norcross, GA, USA) with N_2_ adsorption–desorption analyses. The detailed morphologies of the materials were observed using a scanning electron microscope (Hitachi H-7100, Tokyo City, Japan) and a transmission electron microscope (TEM, JEM2000FXII). Finally, we presented the SEM images of electrodes that were subjected to 100 cycles, dissociated from coin cells, and washed with diethyl carbonate to reveal changes in electrodes during cycling.

### 2.6. Electrochemical Measurements

The electrochemical behavior of the samples was examined using CR2032 coin cells. Pure lithium metal was used as a counter electrode. The working electrode comprised 80 wt.% active material, 10 wt.% super P (carbon black from Taiwan Maxwave Co., Ltd., Taipei City, Taiwan), and 10 wt.% polyvinylidene fluoride. The obtained mixture was dispersed in N-methyl pyrrolidine solution to form a homogeneous slurry. Subsequently, the slurry was pouched onto 10-μm copper foil and dried for 8 h at 120 °C in a vacuum oven. The electrolytes were 1 M LiPF_6_ in 30 wt.% ethylene carbonate and 58 wt.% diethyl carbonate with 2 wt.% vinylene carbonate and 10 wt.% fluoroethylene carbonate. The cells were assembled in a glove box filled with argon, and the H_2_O and O_2_ concentrations were <0.5 ppm. The separators used were of type Celgard 2325. The discharge/charge test results were analyzed using an AcuTech system operated at room temperature and at a voltage window of 0.01 V and 3 V. The electrochemical performance of the working electrodes was investigated through CV (CH Instruments Analyzer CHI 6273E) at a voltage window of 0.01 to 3 V and a scan rate of 0.1 mV·s^−^^1^. Electrochemical impedance spectroscopy measurements were conducted using a CH Instruments Analyzer (CHI 6273E) at a perturbation amplitude of 5 mV and frequency range of 10^5^ Hz to 10 mHz.

## 3. Results and Discussion

**Figure 1:**
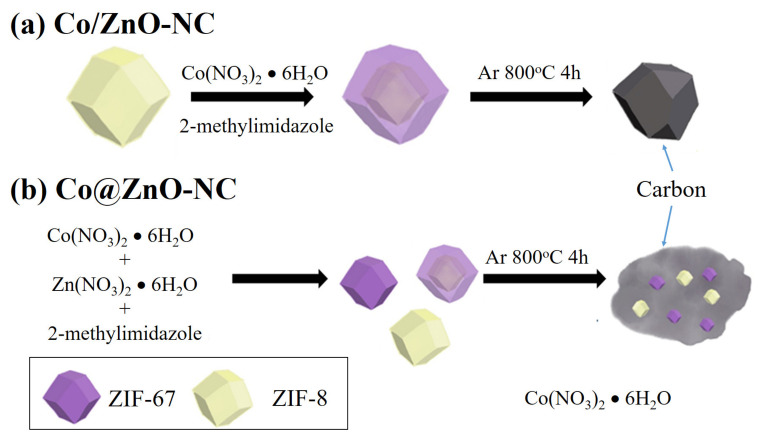
Schematic diagrams of (**a**) Co/ZnO-NC and (**b**) Co@ZnO-NC composite preparation.

Figure 1 presents a schematic of the method for synthesizing porous Co/ZnO-NC and Co@ZnO-NC polyhedral nanostructures. We used ZIF-8 and ZIF-67 as precursors because they have the same topological structure and can be combined with metal cations coordinated with 2-methylimidazole. As indicated previously in this study, we proposed two structural designs of Co@ZnO-NC and Co/ZnO-NC composites. For the Co@ZnO-NC composite, core–shell nanostructures were prepared using a seed-mediated growth method. Subsequently, calcining was conducted for 4 h in an Ar atmosphere at 800 °C. The Co/ZnO-NC composite nanostructures were prepared using Zn(NO_3_)_2_·6H_2_O, Co(NO_3_)_2_·6H_2_O, and 2-methyl imidazole mixed with methanol; calcining was performed under the Ar atmosphere at 800 °C for 4 h.

**Figure 2:**
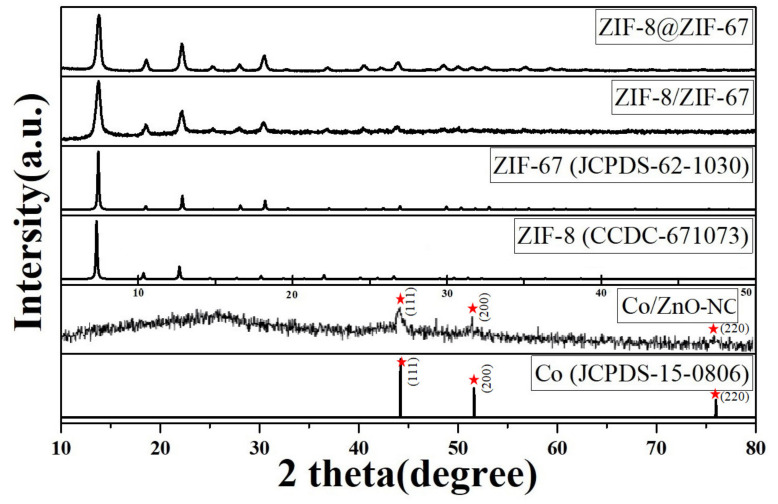
XRD patterns of ZIF-8, ZIF-67, ZIF-8@ZIF-67, ZIF-8/ZIF-67, Co/ZnO-NC, and Co. The red stars in the Figure is Co.

The crystal phase structures of the ZIF-8@ZIF-67 and ZIF-8/ZIF-67 composites are presented as XRD patterns (Figure 2). The diffraction peaks (2θ) of 7.31°, 10.57°, 12.85°, 14.88°, 16.72°, and 18.3° corresponded to the peaks of (100), (002), (112), (022), (013), and (222), respectively. The XRD patterns of ZIF-8@ZIF-67 and ZIF-8/ZIF-67 matched the simulated patterns of ZIF-8 (JCPDS-62-1032) and ZIF-67(CCDC-671073), respectively. The strong and sharp profiles of these diffraction peaks confirmed high crystallinity. Figure 2 presents the XRD patterns of Co/ZnO-NC. The diffraction peaks (2θ) of 44.2°, 51.6°, and 75.9° corresponded to the peaks of (111), (200), and (220), respectively. The results indicated that Co (Marked by red star) was produced after the calcination of ZIF-8/ZIF-67. The broad peak at approximately 26° was attributed to the (002) peak of graphitic carbon.

**Figure 3:**
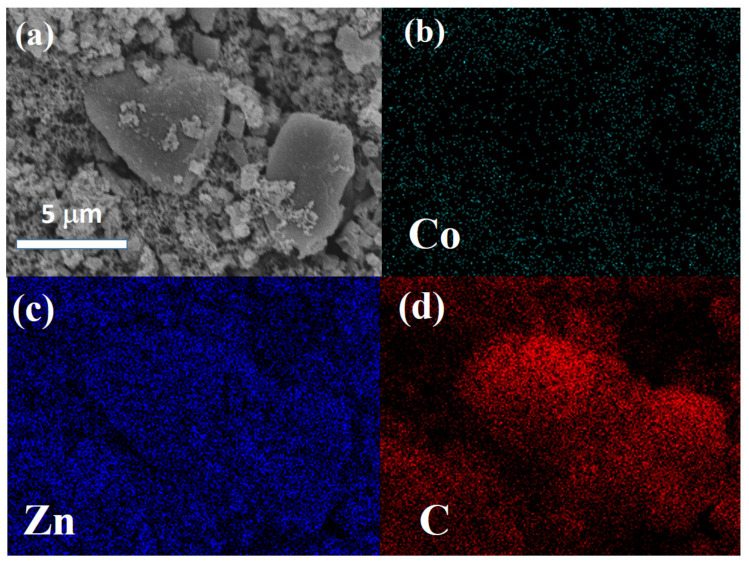
(**a**) SEM and EDS mapping of (**b**) Co, (**c**) Zn, and (**d**) C of Co/ZnO-NC.

**Figure 4:**
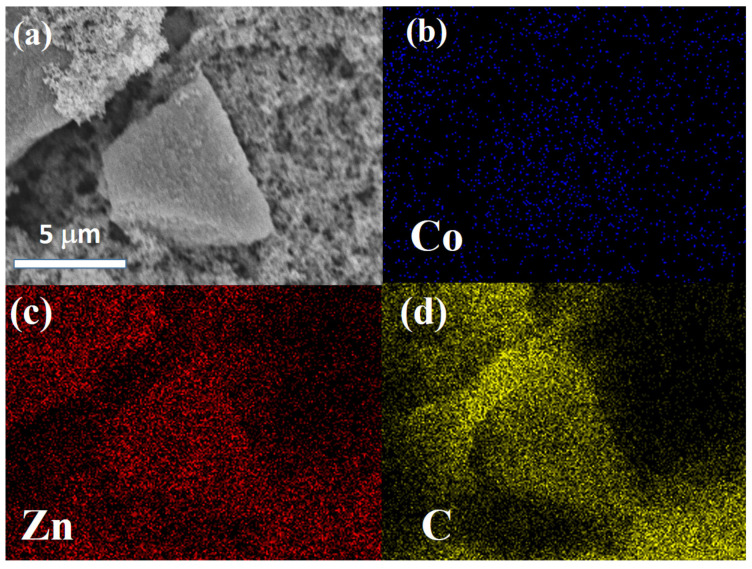
(**a**) SEM and EDS mapping of (**b**) Co, (**c**) Zn, and (**d**) C of Co@ZnO-NC.

Figure 3 and Figure 4 show SEM images and the corresponding EDS mapping of Co, Zn, and C for Co/ZnO-NC and Co@ZnO-NC, respectively. As shown in Figure 3, Co and Zn were homogeneously distributed in carbon matrix. Due to the composite-type Co/ZnO-NC structure, there was no obvious aggregation in Figure 3a. Figure 4a displays the surface morphology of core-shell type Co@ZnO-NC sample. More aggregated particles with the particle size of ~10 mm was observed in Figure 4a. Compared to Figure 3b, the signal of Co was more obvious for Co/ZnO-NC sample. It was due to the core-shell structure was successfully constructed by our process. The elemental distribution of Zn and C of Co/ZnO-NC and Co/ZnO-NC are shown in Figure 3c and Figure 3d, respectively. Both Zn and C were also well-distributed in these two composite. The results of Figure 4b–d supported that Co@ZnO was homogeneously embedded into carbon matrix. 

**Figure 5:**
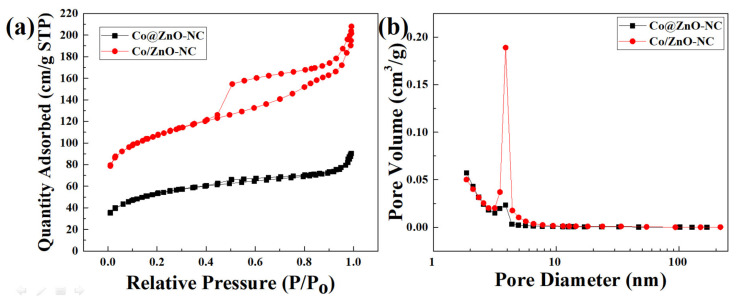
(**a**) N_2_ adsorption isotherms and (**b**) pore size distributions of Co@ZnO-NC and Co/ZnO-NC.

Figure 5a,b presented the nitrogen adsorption/desorption isotherms and pore size distributions of as-synthesized Co/ZnO-NC and Co@ZnO-NC composites. As indicated in Figure 5a, the Co/ZnO-NC sample had typical type IV features with a distinct hysteresis loop at a high P/P_0_ of 0.4–1.0. The average pore size of Co/ZnO-NC composite was 3.87 nm. The average pore size of Co@ZnO-NC was 3.04 nm, and the specific surface area was 177.03 m^2^/g. These findings indicated the existence of many micropores and mesopores in these materials. The specific surface area of Co/ZnO-NC was 350.53 m^2^/g; it had a larger specific surface area and more mesopores than Co@ZnO-NC. The large specific surface area of the mesoporous structure was conducive to adaption to volumetric changes caused by Li ion extraction and insertion; therefore, the Co/ZnO-NC composite may provide more active sites for lithium storage compared with the Co@ZnO-NC composite.

**Figure 6:**
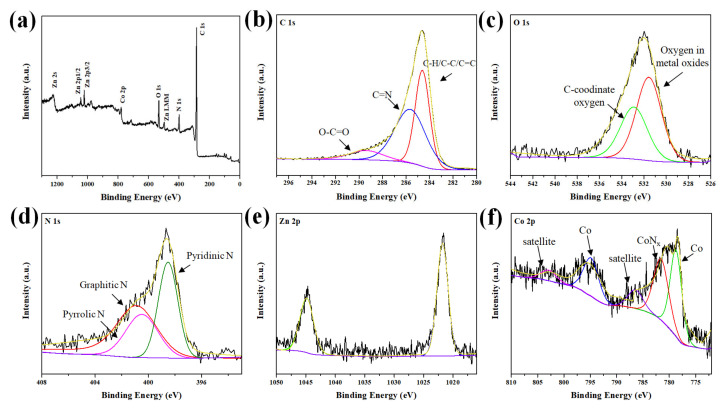
XPS spectra of Co/ZnO-NC: (**a**) survey scan spectrum, (**b**) high-resolution C 1s spectrum, (**c**) O 1s spectrum, (**d**) N 1s spectrum, (**e**) Zn 2p spectrum, and (**f**) Co 2p spectrum.

An XPS survey of the Co/ZnO-NC composite, as indicated in Figure 6a, was conducted to identify its surface composition and oxidation state. The results confirmed the presence of C, N, O, Co, and Zn. A high-resolution XPS spectrum of the Co/ZnO-NC composite for C 1s is displayed in Figure 6b. The spectrum could be deconvoluted into four bands as follows: C-H/C-C/C=C at 284.6 eV, C-O at 286.2 eV, C=N at 285.7 eV, and O-C=O at 289.6 eV. The O 1s spectrum, as indicated in Figure 6c, exhibited three oxygen bonding features. The peak at 531.5 eV was typical of a metal–oxygen bond, and the peak at 532.9 eV was associated with oxygen-functionalized carbon, chemisorbed oxygen, and under-coordinated lattice oxygen. This result indicated that the oxidation process induced the formation of metal oxides and a disordered carbon phase. The N 1s spectrum presented in Figure 6d could be deconvoluted into three peaks at 398.5, 399.8, and 401.2 eV, corresponding to pyridinic, pyrrolic, and graphitic N atoms, respectively. The proportions of pyridinic, pyrrolic, and graphitic N in Co/ZnO-NC were 33.4%, 32.8%, and 33.7%, respectively. N atoms originating from imidazole moieties were doped into the composite. In addition, pyridinic N atoms were strongly set on both graphitic carbon and metal atoms, thus considerably improving structural stability. The peaks of Zn 2p, as presented in Figure 6e, exhibited two obvious bands at 1021.7 and 1044.7 eV, which could be assigned to Zn 2p3/2 and Zn 2p1/2, respectively. This result suggested that Zn in the composites was converted to ZnO. For Co 2p, as shown in Figure 6f, two distinct peaks were observed at 779.5 and 796.2 eV. These two peaks could be assigned to Co^0^ [39]. This result confirmed that N doping into the carbon frameworks of Co/ZnO-NC was achieved; this achievement is expected to improve the electrochemical performance of LIBs.

**Figure 7:**
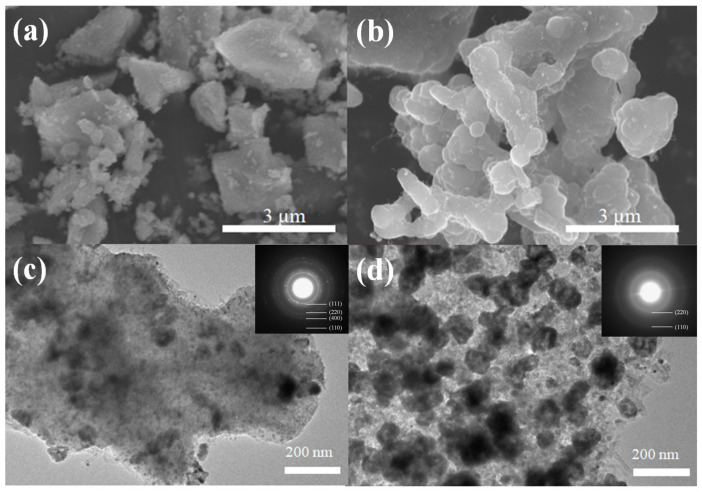
(**a**) SEM and (**c**) TEM images of Co/ZnO-NC; (**b**) SEM and (**d**) TEM images of Co@ZnO-NC. Insets in (**c**,**d**) are the corresponding selected area electron diffraction patterns.

Figure 7a,b presented the representative SEM images of Co/ZnO-NC and Co@ZnO-NC. As indicated, both powders were aggregates; however, their surfaces still had many fiber-like structures on the surface. According to the literature [39], in the presence of inert gases, Co nanoparticles can act as a catalyst for CNTs (carbon nanotubes) production. CNTs can be generated in situ under the catalytic effect of cobalt nanoparticles at a high temperature and with sufficient partial pressure of Ar. TEM images of Co/ZnO-NC and Co@ZnO-NC are presented in Figure 7c,d, respectively. The insets of Figure 7c,d display the corresponding selected area electron diffraction patterns. Figure 7c indicates that the diffraction rings of Co/ZnO-NC conformed to the (220), (111), and (110) planes of Co and with the (400) planes of ZnO. Figure 7d reveals that the diffraction rings of Co/ZnO-NC are in favorable agreement with the (220), (111), and (110) planes of Co. The results related to Co@ZnO-NC indicated that Co was encapsulated on the surface of Co@ZnO-NC composite. These crystal planes of the composite match the XRD measurements presented in Figure 2. The TEM images of Co/ZnO-NC composite indicated that the Co and ZnO-NC were mixed homogeneously. Many small tubular fibers formed around the structure of the composite, a finding that is consistent with the SEM results. The carbon nanotubes growing on the surface of the Co/ZnO-NC composite may increase its surface area and facilitate the rapid migration of lithium ions, effectively improving the electrochemical performance of LIBs.

**Figure 8:**
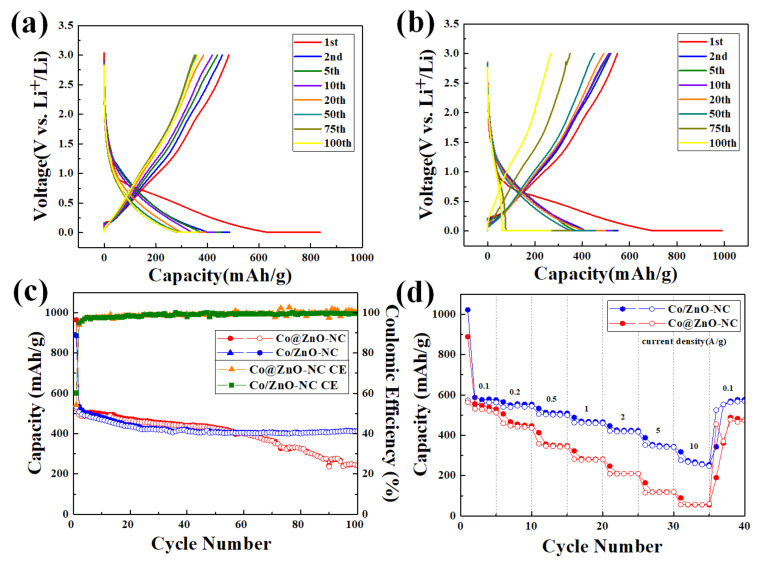
Charge/discharge curves of (**a**) Co/ZnO-NC and (**b**) Co@ZnO-NC at 200 mA/g; (**c**) Cycling performance of Co@ZnO-NC and Co/ZnO-NC at 200 mA/g; (**d**) Rate performance of Co@ZnO-NC and Co/ZnO-NC.

Figure 8a,b presented the charge/discharge curves of Co@ZnO-NC and Co/ZnO-NC samples at a current density of 200 mA/g over 100 cycles. The discharge capacity of Co@ZnO-NC at the 1st, 2nd, 3rd, 5th, 10th, 20th, 50th, 75th, and 100th cycle was 836, 485, 450, 430, 393, 359, 353, and 363 mAh/g, respectively. The corresponding charge capacity at the 1st, 2nd, 3rd, 5th, 10th, 20th, 50th, 75th, and 100th cycle was 482, 457, 438, 418, 385, 356, 350, and 361 mAh/g, respectively. The corresponding Coulombic efficiency of Co@ZnO-NC was 57%, 94%, 97%, 97%, 97%, 99%, 100%, and 99% at the 1st, 2nd, 3rd, 5th, 10th, 20th, 50th, 75th, and 100th cycle, respectively. The discharge capacity of Co/ZnO-NC at the 1st, 2nd, 3rd, 5th, 10th, 20th, 50th, 75th, and 100th cycle was 989, 550, 528, 523, 496, 454, 354, and 266 mAh/g, respectively. The charge capacity of Co/ZnO-NC at the 1st, 2nd, 3rd, 5th, 10th, 20th, 50th, 75th, and 100th cycle was 548, 519, 516, 512, 490, 450, 348, and 267 mAh/g, respectively. The Coulombic efficiency of Co/ZnO-NC was 55%, 94%, 97%, 97%, 98%, 99%, 98%, and 100% at the 1st, 2nd, 3rd, 5th, 10th, 20th, 50th, 75th, and 100th cycle, respectively. A large irreversible capacity at the first cycle was ascribed to the solid electrolyte interface layer (SEI) and the initial irreversible reactions of the active materials. Figure 8c shows the cycling stability of the Co@ZnO-NC and Co/ZnO-NC for 100 cycles at 200 mA/g. Both exhibited stable cycle stability during the first 50 cycles. The discharge/charge capacities of Co@ZnO-NC and Co/ZnO-NC after 100 cycles were 411 and 246 mAh/g, respectively. The capacity of Co@ZnO-NC slightly decreased from 498 mAh/g to 246 mAh/g after 100 cycles, presumably because the damage to the internal structure was more severe compared to Co@ZnO-NC composite. In the first cycle, the Coulombic efficiency of Co/ZnO-NC was as low as 52%; this could be ascribed to the chemical reaction of the electrolyte and the formation of the SEI layer. The capacity retention rates of Co@ZnO-NC and Co/ZnO-NC were 49% and 82%, respectively. Figure 8d reveals that the rate performance levels of Co/ZnO-NC and Co@ZnO-NC were measured at the current densities of 0.1, 0.2, 0.5, 1, 2, 5, and 10 A/g before the current density was reverted to 0.1 A/g. Co/ZnO-NC had an average specific capacity of 572, 548, 503, 460, 417, 345, 257, and 567 mAh/g, respectively, and Co@ZnO-NC had an average specific capacity of 531, 455, 350, 276, 207, 117, 52, and 476 mAh/g, respectively. Unlike the Co/ZnO-NC electrode, the reversible specific capacity of Co@ZnO-NC decreased as the current density increased. This further demonstrated that Co/ZnO-NC is superior to Co@ZnO-NC as an anode for LIBs. The superior conductivity and abundant porous channels of Co/ZnO-NC may provide an intrinsic guarantee of the fast transport of electrons and Li^+^ within electrodes.

**Figure 9:**
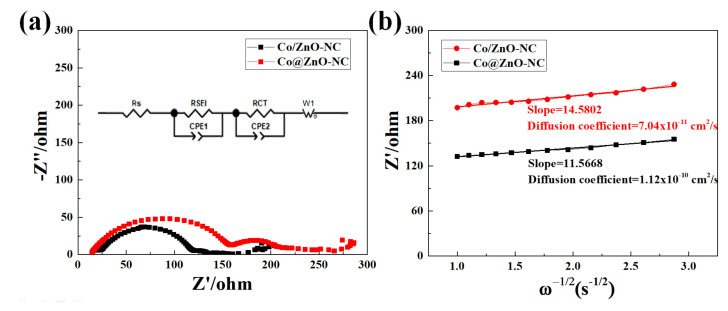
(**a**) Nyquist plots for Co@ZnO-NC and Co/ZnO-NC electrodes; (**b**) Corresponding Randles plots for Co@ZnO-NC and Co/ZnO-NC electrodes.

Figure 9a presents AC impedance measurement results for Co/ZnO-NC and Co@ZnO-NC electrodes; these measurements were undertaken to understand these electrodes’ lithium storage properties. Each Nyquist plot can be approximately divided into many sections. The high-frequency intercept on the axis represents electrolyte resistance (R_s_). The depressed semicircle at medium frequencies generally relates to charge transfer resistance (R_CT_), the SEI (Solid electrolyte interphase) layer (R_SEI_), and the slope at a low frequency represents Warburg impedance in relation to the diffusion of the lithium ion process [45]. The larger the diameter of the semicircle is, the greater is the charge transfer resistance. The fitting result is shown in Table 1. The R_SEI_ of Co@ZnO-NC and Co/ZnO-NC was 144 Ω and 97 Ω, respectively. The R_CT_ of Co@ZnO-NC and Co/ZnO-NC was 49 Ω and 15 Ω, respectively. The R_CT_ and R_SEI_ of the Co/ZnO-NC were considerably smaller than those of Co@ZnO-NC, indicating that Co/ZnO-NC electrodes have smaller resistance than core–shell electrodes. As indicated in Figure 7b, the Li ion diffusion coefficient of these two samples can be calculated using the following equation:

(1) $$D_{\mathrm{Li}}=\frac{R^{2}T^{2}}{2A^{2}n^{4}F^{4}C^{2}\sigma^{2}}$$

where D_Li+_ is the diffusion coefficient of lithium ion. R (8.314 J/mol × K) is the gas constant, T is the absolute temperature (298.15 K in the present study), n is the number of electrons per molecule during oxidization (n = 1), F (96,485.3 C mol^−1^) is the Faraday’s constant, A is the area of the anode/electrolyte interface, C (0.001 mol cm^−^^3^) is the concentration of lithium ions, and σ is the Warburg factor, which is related to Z_re_ [46]. Using slope values σ, as shown in Figure 9b, the diffusion coefficients of Co/ZnO-NC and Co@ZnO-NC were determined to be 7.04 × 10^−11^ and 1.12 × 10^−10^ cm^2^/s, respectively. The results indicated that the Co/ZnO-NC electrode had higher Li ion diffusivity than did the other electrode. These results confirmed the excellent cycling stability and electrical conductivity of Co/ZnO-NC composites.

**Figure 10:**
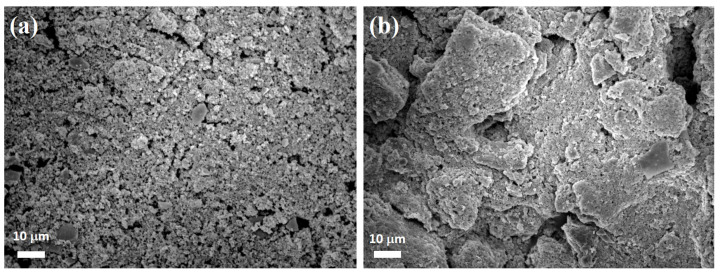
SEM images of Co/ZnO-NC electrodes before (**a**) and after cycling (**b**).

Figure 10a,b showed the SEM images of Co/ZnO-NC electrodes before and after cycling tests, respectively. As shown in the SEM images, even for 100 cycles, the surface morphology of Co/ZnO-NC electrode remained condense structure without cracking. The result indicated that composite-type Co/ZnO-NC demonstrate an excellent structural stability during charge and discharge processes.

**Figure 11:**
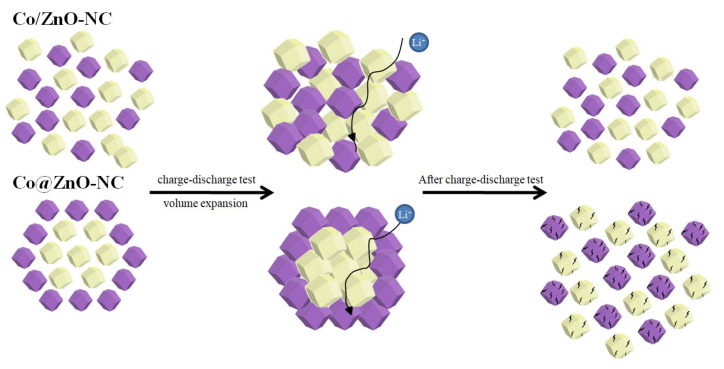
Schematic of Co/ZnO-NC and Co@ZnO-NC electrodes before and after discharge/charge tests.

Figure 11 presents a schematic of the charge and discharge process of Co/ZnO-NC and Co@ZnO-NC. Co@ZnO-NC expanded and shrank after many charge and discharge cycles such that the cobalt covered by the outer layer collapsed, resulting in poor electrochemical properties. The structure of Co/ZnO-NC was not covered, and its structural composition was not altered during charge and discharge processes. The electrochemical performance of Co/ZnO-NC was more favorable than that of Co@ZnO-NC.

## 4. Conclusions

In this study, two blending methods and high-temperature sintering were used to produce Co/ZnO-NC and Co@ZnO-NC. The porous structure of MOFs is conducive to Li ion transfer, and a high temperature enabled the production of nitrogen-doped carbon and fibrous carbon nanotubes on the surfaces of these materials. Electrical analyses demonstrated that Co/ZnO-NC exhibited higher cycling stability and reversible capacity than Co@ZnO-NC. According to the rate capability tests, Co/ZnO-NC exhibited higher rate capability. The diffusion coefficients of lithium ions in Co/ZnO-NC and Co@ZnO-NC were 7.04 × 10^−^^11^ and 1.12 × 10^−^^10^ cm^2^/s, respectively. High electrochemical performance can be obtained by blending ZIF-67 and ZIF-8 precursors. The results revealed that the Co/ZnO-NC composite is a potential anode material for LIBs.

## Figures and Tables

**Table 1:** R_SEI_, R_CT_, and diffusion coefficient of Li in Co/ZnO-NC and Co@ZnO-NC electrodes.

Components	Co/ZnO-NC	Co@ZnO-NC
Rs (Ω)	23	18
R_SEI_ (Ω)	97	144
R_CT_ (Ω)	15	49
σ (Ω/s^0.5^)	14.58	11.56
D (cm^2^/s)	7.04 × 10^−11^	1.12 × 10^−10^

## Data Availability

Not applicable.
